# Diffusion MRI of Structural Brain Plasticity Induced by a Learning and Memory Task

**DOI:** 10.1371/journal.pone.0020678

**Published:** 2011-06-20

**Authors:** Tamar Blumenfeld-Katzir, Ofer Pasternak, Michael Dagan, Yaniv Assaf

**Affiliations:** 1 Department of Neurobiology, The George S. Wise Faculty of Life Sciences, Tel Aviv University, Tel Aviv, Israel; 2 Laboratory of Mathematics in Imaging, Department of Radiology, Brigham and Women's Hospital, Harvard Medical School, Boston, Massachusetts, United States of America; Louisiana State University Health Sciences Center, United States of America

## Abstract

**Background:**

Activity-induced structural remodeling of dendritic spines and glial cells was recently proposed as an important factor in neuroplasticity and suggested to accompany the induction of long-term potentiation (LTP). Although T1 and diffusion MRI have been used to study structural changes resulting from long-term training, the cellular basis of the findings obtained and their relationship to neuroplasticity are poorly understood.

**Methodology/Principal Finding:**

Here we used diffusion tensor imaging (DTI) to examine the microstructural manifestations of neuroplasticity in rats that performed a spatial navigation task. We found that DTI can be used to define the selective localization of neuroplasticity induced by different tasks and that this process is age-dependent in cingulate cortex and corpus callosum and age-independent in the dentate gyrus.

**Conclusion/Significance:**

We relate the observed DTI changes to the structural plasticity that occurs in astrocytes and discuss the potential of MRI for probing structural neuroplasticity and hence indirectly localizing LTP.

## Introduction

Neuroplasticity can be defined as experience-dependent structural or functional changes in neurons. The hallmark processes of neuroplasticity in the adult brain are induction of long-term potentiation (LTP) [1], [2], neurogenesis [3], [4], [5], and structural remodeling of various cellular components [1], [2], [6], [7], [8], [9], [10], [11]. Structural remodeling of tissue, or ‘structural plasticity’, refers to changes in the shape and number of cellular structures in response to a continuous demand for a specific activity [1], [2], [6], [7]. Such an activity could be the formation of new neurons (neurogenesis) [12], [13], new synapses (synaptogenesis) [1], [2], [6], or additional dendritic branches (dendrogenesis) [14]; axonal or synaptic sprouting [15]; change in the shape or number of glial cells (gliogenesis), especially of astrocytes [8], [16], [17], [18]; and even changes in the oligodendrocytes and myelin formation (white matter plasticity) [19], [20], [21], [22].

Magnetic resonance imaging (MRI) offers a wide variety of anatomical measures. Although regarded as a high-resolution imaging modality, its resolution in the context of brain plasticity is far below the cellular level. Despite its low specificity and resolution limits, however, T_1_ MRI contrast has been used to estimate cortical density, compare it before and after training episodes, and correlates it with specific cognitive skills [23], [24], [25]. Those studies have suggested that regional changes in cortical density may reflect structural plasticity. Thus, for example, the results of a cross-sectional study of the hippocampi of taxi drivers, and findings from a follow-up study of individuals trained to juggle [23], [24], [25], point to significant tissue changes in the adult brain following long-term training, thereby shedding new light on the process of plasticity and also raising new questions about it.

Diffusion MRI in general, and diffusion tensor imaging (DTI) in particular [26], [27], is regarded as a microstructural probe [28], [29]. As such, it might provide more specific information on plasticity-related morphological tissue changes than conventional anatomical MRI [30]. The various indices extracted from DTI enhance image specificity for distinct brain structures (e.g. fractional anisotropy (FA) is used to characterize the organization of white matter fibers) [26], [31], [32]. Significant changes in DTI parameters were reported in the relevant white matter pathways after training [21], [33], [34], leading to speculation that DTI can detect structural brain plasticity in both gray and white matter [30]. However, the biological and morphological meanings of these changes remain unclear.

In this study we examined rats before and after a task involving learning and memory, and employed voxel-based statistics to compare the induced brain changes as reflected by DTI indices. As controls for the experimental group of rats that were trained for the learning/memory task (group L), the same analysis was applied to rats that performed a task that required the same motor behavior (swimming) but did not involve learning and memory (group S), as well as to rats that did not undergo behavioral manipulation (group NL). In addition, we investigated the age dependency of the plasticity changes by analyzing induced brain alterations after performance of the task by rats of different ages. The MRI results were compared with histological findings. Based on our results, we discuss the potential of diffusion MRI to assess *in-vivo* multi-regional brain plasticity both in white matter and in gray matter.

## Results

### Behavioral results

To induce plasticity related to learning- and memory we trained rats aged 1, 4, and 12 months on the Morris water maze task over 5 days. Rats of all three ages in the experimental (learning and memory) group (“group L”) showed significant improvements on each successive day, evidenced by an ongoing decrease in latency (Fig. S1). Although all rats in each age subgroup successfully performed the task and exhibited pronounced spatial learning effects, the younger groups (aged 1 or 4 months) outperformed the older group (aged 12 months).

### Volumetric analysis

To evaluate volumetric changes that occurred as a result of the water maze task, the hippocampus was segmented three-dimensionally from the various DTI maps (following registration only) using a multi-dimensional approach [35] with the FA and principal diffusivities as a multi-dimensional dataset input. The hippocampal volume was calculated from the segmented maps and the pre- and post-MRI volumes were compared. The post-MRI increase in volume was slight and nonsignificant (<1%, *P* = 0.07; data not shown).

### Effects of learning and memory on DTI indices

**Figure 1:**
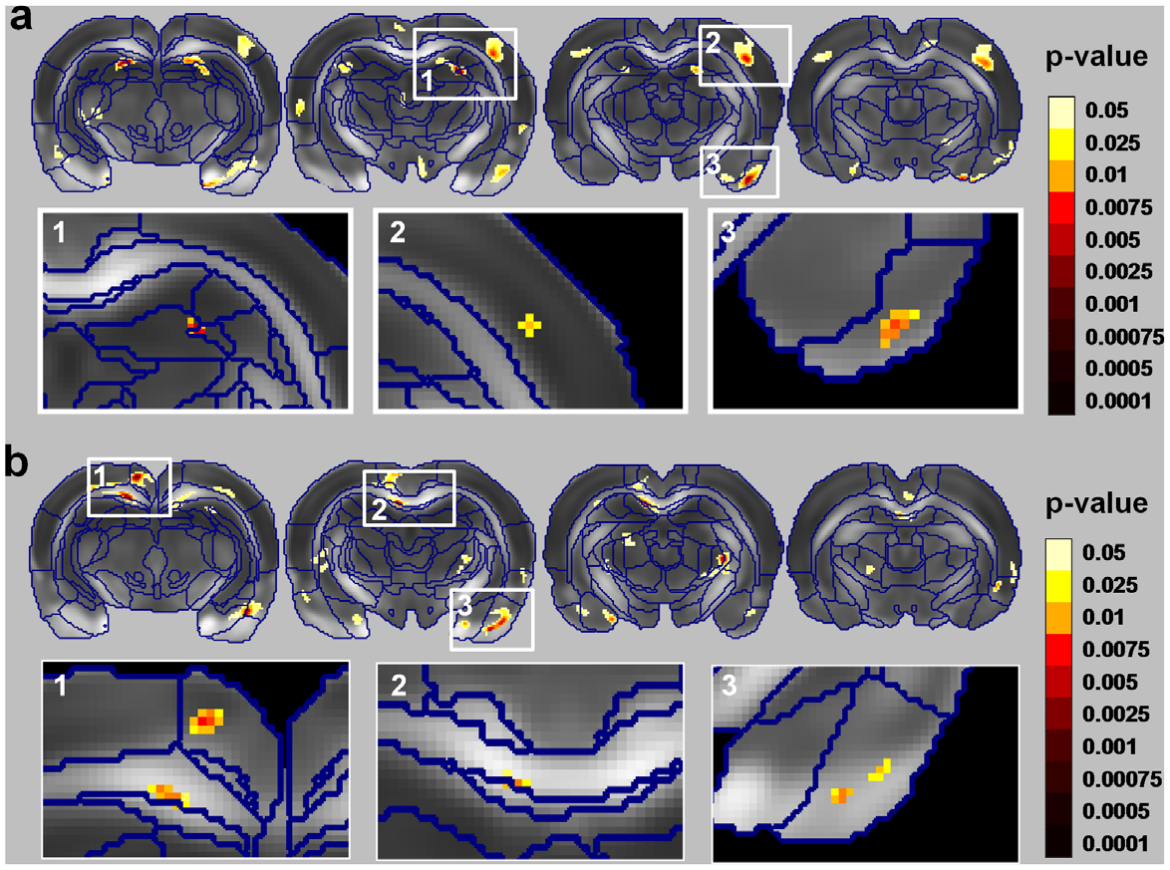
Statistical parametric maps of the interactions between scan time and study group [learning (L), swimming only (S) and nonlearning (NL)] for (a) the apparent diffusion coefficient (ADC) and (b) the fractional anisotropy (FA). The statistical maps (colored regions) are superimposed on an averaged FA map of all rats that were scanned, with borders of the different anatomical regions outlined in blue (see Methods). Voxels that exceed a statistical threshold of *P*<0.05 (non-corrected) are colored according to the threshold they exceeded (see color scale), while those that did not exceed the threshold are not colored. We report only on regional clusters that exceeded a statistical threshold of *P*<0.05 corrected for multiple comparisons; these regions are shown in the insets. These regions include the dentate gyrus (DG), piriform cortex (PC), and S1/S2 cortex (SC) in the ADC maps (**a**), and the corpus callosum (CC) and PC in the FA maps (**b**).

Voxel-based mixed-design analysis of variance (ANOVA) between the two factors, group (learning [L], swimming only [S], and nonlearning [NL]) and time (1^st^ and 2^nd^ MRI scans), revealed significant multiregional interaction, indicating that in certain brain regions changes in DTI indices differed between the three groups (Fig. 1) as a function of scanning time. This was observed for both the apparent diffusion coefficient (ADC) and the fractional anisotropy (FA) in several brain regions, including the: dentate gyrus (DG), cingulate cortex (CG, posterior part), piriform cortex (PC), S1/S2 cortex (SC) and corpus callosum (CC). These regions exceeded a statistical threshold of *P*<0.05 (corrected for multiple comparisons); nevertheless, for visualization purposes figure 1 also shows voxels that exceeded a non-corrected threshold (*P*<0.05). Similar analysis was performed on the radial and axial diffusivities (see supporting text S1 and Figure S2).

**Figure 2:**
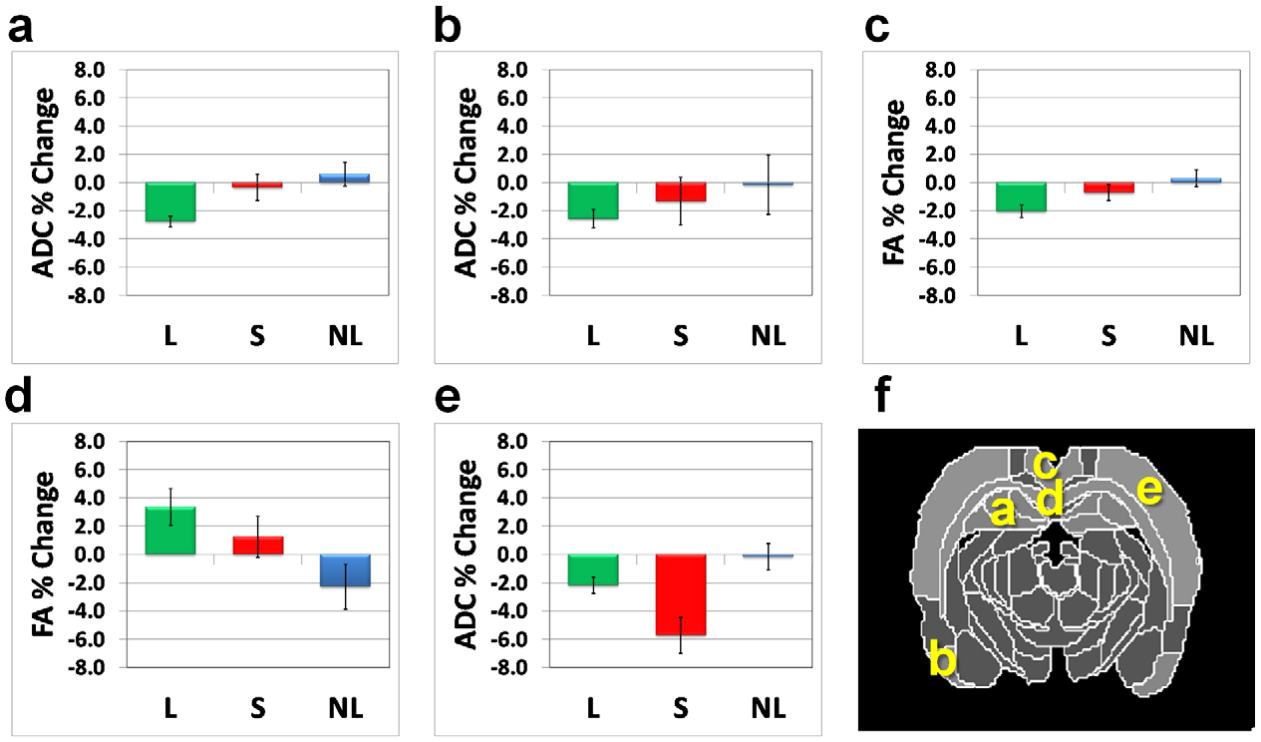
Regional analysis of the parametric interaction maps. Type 1 of interaction, i.e., regions that exhibit significant changes in the learning group (L) only were identified in ADC in the dentate gyrus (**a**) and piriform cortex (**b**) and FA in the cingulate cortex (**c**) and corpus callosum (**d**). Type 2 of interaction, i.e., regions that exhibit significant changes in the swimming-only (S) group and to lesser extent in the learning (L) group was found in the S1/S2 cortex (**e**). (**f**) Brain atlas diagram with the abovementioned regions indicated.

Regional analysis of each of the ANOVA interaction clusters was used to identify the study groups contributing to the interaction. The analysis yielded two types of interactions. In the first, group L contributed to the interaction with no apparent changes in groups S and NL. In the second, group S contributed to the interaction with no apparent changes in groups L or NL. Figure 2 presents the regional analysis for the two types of interaction. The first included a decrease in ADC (2%–3%) in the DG (Fig. 2a) and PC (Fig. 2b); a decrease in FA (2%–3%) in the CG (Fig. 2c), and an increase in FA (2%–4%) in the CC (Fig. 2d). The second included a decrease in ADC (∼6%) in the SC (Fig. 2e).

### Comparison of MRI with histology findings

Histological analysis was utilized in an attempt to relate the structural basis of neuroplasticity, as observed on DTI, to its cellular basis. The DTI findings were compared with immunohistochemical analyses that were based on staining by a neuronal marker (NeuN), a synaptic marker (synaptophysin), a dendritic marker (MAP2), a myelin marker (MBP), and an astrocyte marker (glial fibrillary acidic protein; GFAP). Two regions were compared: the dentate gyrus (DG) of the hippocampus and the corpus callosum (CC). We chose these regions as markers of areas exhibiting a decrease in ADC (the DG) and an increase in FA (the CC) in group L.

**Figure 3:**
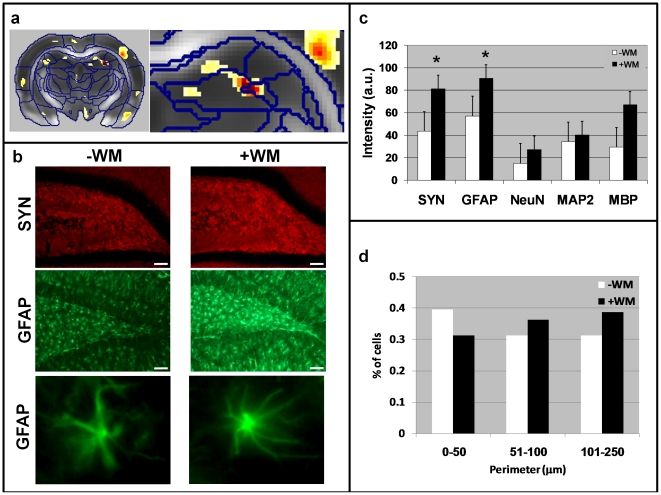
Geometrical and histological analyses of the dentate gyrus and hippocampus in representative mice from group L. (**a**) Statistical parametric map of interaction between scan time and group in a representative slice that includes the hippocampus and dentate gyrus (DG) region. An enlargement of this region is shown on the right. (**b**) Immunohistochemical staining (×10 magnification) of the cell layer of the DG and hippocampal hilus for synaptophysin (SYN, a marker of synapses) in red, and glial fibrillary acidic protein (GFAP, a marker of astrocytes) in green. Note the increase in SYN and GFAP immunoreactivity in the hilus of the DG after completion of the water maze task. GFAP immunoreactivity in the cellular layer of the DG was also increased following the task. The white bar at the bottom right corner represents 100 µm. The bottom panel of (**b**) shows enlargements of two representative astrocytes, demonstrating the massive structural changes that occurred in this cell type in this study group. (**c**) Quantification of the immunoreactivity (staining intensity) in the hilus of the DG for different markers shows significant increases in SYN and GFAP staining. Asterisk (*) denotes *P*<0.05. (**d**) Histogrammatic analysis of the perimeters of GFAP-stained cells in the hilus of the hippocampus. After completion of the water maze task there were fewer astrocytes with small perimeters and more astrocytes with large perimeters indicative of more numerous processes.

For the DG comparison, we stained the hilus of the dentate gyrus (Fig. 3). Of the abovementioned cellular markers, only those marking synapses and astrocytes showed a significant increase in staining intensity (Fig. 3b). Geometrical analysis of astrocyte shape revealed an increase in both the volume (Fig. S3) and the perimeter of these cells (Fig. 3d). This observation was further supported by images of representative cells pointing to a dramatic increase in the number of astrocytic processes in the water maze-trained rats compared with naïve rats (Fig. 3b, bottom panel). This is in line with the observed decrease in ADC, as an increase in the number of cellular processes would be expected to lead to an increase in tissue density.

**Figure 4:**
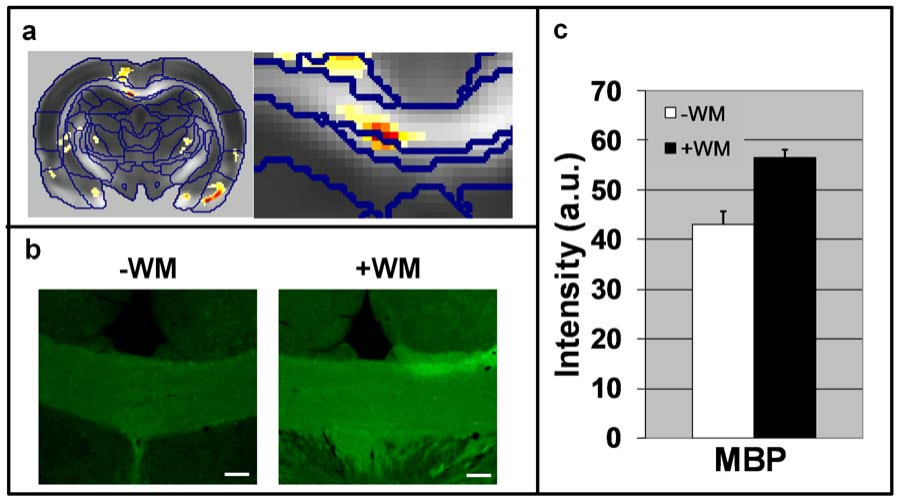
Histological analysis of the corpus callosum in representative mice from group L. (**a**) Statistical parametric maps of interaction between scan time and group in a representative slice that includes the corpus callosum (CC). An enlargement of this region is shown on the right. (**b**) Immunohistochemical staining (×10 magnification) of the CC for MBP. Note the increase in MBP immuno-reactivity in the CC after completion of the water maze task. The white bar at the bottom right corner represents 100 µm. (**c**) Quantification of the immunoreactivity (staining intensity) in the CC. Asterisk denotes *P*<0.05.

The CC comparison is depicted in Figure 4. Here the only cellular marker showing significant changes in immune reactivity was MBP. This result indicates that the oligodendrocytes forming the myelin sheaths produced more MBP, probably to support the required flow of information. This is in line with the increase in FA in this region, possibly implying an increase in the cellular organization and packing of axons or myelin.

### Plasticity and aging

**Figure 5:**
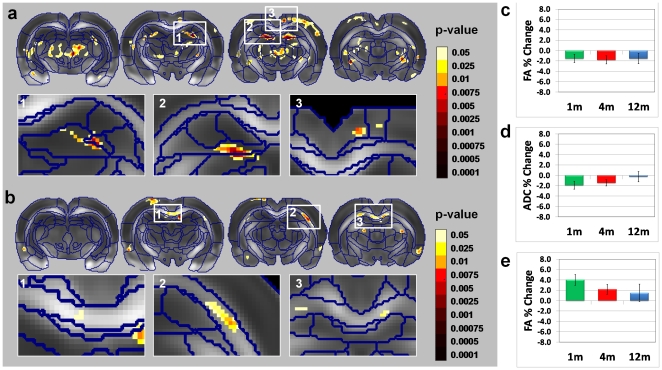
Age dependency of the changes in diffusion tensor imaging induced by spatial learning. (**a**) and (**b**) Statistical parametric maps of the scan time main effect over the three group L subgroups (rats that underwent the water maze task at the age of 1, 4, or 12 months) for ADC and FA, respectively. Voxels that exceed a statistical threshold of *P*<0.05 (non-corrected) are colored according to the threshold they exceeded (see color scale), while those that did not exceed the threshold are not colored. We report only on regional clusters that exceeded a statistical threshold of *P*<0.05 corrected for multiple comparisons (using the FDR method); these regions are shown in the insets. The regional pattern of the main effect (in both the ADC and the FA maps) includes the dentate gyrus (DG) and cingulate cortex as well as the corpus callosum. Post-hoc analysis of the main effects revealed that in some regions the magnitude of DTI changes between the two scans was similar across ages. Examples are reduction in ADC in the in the DG (**c**). Other regions show an age-dependent relationship in which effects were more pronounced in younger than in older mice. This was observed in the CG (**d**) and the corpus callosum (**e**).

In the following analysis we probed the age dependency of changes in DTI indices induced by learning and memory. This was done by ANOVA with two factors: age (1, 4, and 12 months) and scan time (1^st^ and 2^nd^ scans) with repeated measures on the second factor. Results of the main effect of scan time over the three age subgroups are given in Figure 5 for both ADC and FA. The regions that exceeded the statistical threshold for the scan/time main effect (Fig. 5) were similar to the regions found in the interaction effect between the learning and the control groups at 4 months (Fig. 1). These results indicate that the regional pattern of DTI changes induced by learning and memory is not altered with age. Regional analysis revealed that in some regions (DG) the effect was similar in magnitude for all age groups (Fig. 5c), while in others (CG) the effect was stronger for the younger groups (Fig. 5d) and decreased with age. In addition, changes in FA in white matter regions (CC) were more pronounced in the younger groups (Fig. 5e) and decreased with age.

## Discussion

Activity-dependent structural remodeling of dendritic spines and adjacent glial processes has been suggested to play a significant role in the induction of LTP, plasticity and memory [8], [9], [10], [36], [37]. Support for this hypothesis comes from post-mortem histological or invasive *in-vivo* animal studies. The main finding of this MRI study is that diffusion MRI can be used to assess structural plasticity, which occurs to a significant extent in the adult brain and extends from the region of the hippocampus and dentate gyrus to other cortical and sub-cortical regions. Our results suggest that diffusion MRI can be used to visualize structural remodeling and thereby indirectly localize LTP and plasticity.

### Localization of plasticity induced by learning and memory

The Morris water maze task used in this study to test learning and memory appears to induce significant changes in DTI indices in the dentate gyrus as well as in various regions of the limbic system (i.e. cingulate cortex, dentate gyrus, and piriform cortex) and white matter systems (corpus callosum). The DTI changes observed in the learning group of rats were in line with the functional localization of this task, underscoring the usefulness of diffusion MRI in general and DTI in particular as an *in-vivo* probe of structural plasticity in the brain.

A surprising result was the localization of brain changes in the control groups. The swimming-only group showed significant changes in sensory related regions (S1/S2 cortex) that were not apparent in the learning or the nonlearning groups. This was despite the fact that the learning group, in addition to memorizing the location of the platform, performed the same motor function as the swimming-only group and might therefore be expected to show similar changes in the S1/S2 cortex. This finding might indicate an attention shift in the learning group so that most of the functional effort in these rats was localized in the limbic areas, leading to much smaller structural changes in sensory/motor regions (fig. 2e). When the task did not involve spatial memory (as in the swimming-only group) most of the functional effort was apparently concentrated in sensory/motor-related areas, leading to changes in those regions.

### Age dependency

Following the spatial navigation task, the localization of structural changes as indicated by diffusion MRI seemed to be independent of age (Fig. 5). However, the magnitude of the effect varied between regions. Accumulating evidence suggests that structural plasticity extends beyond the regions of the hippocampus in adult brain. Our results support this notion, as we showed that these structural changes occur in numerous regions of the rat brain region even in older animals (aged 12 months).In addition, at least some of these regions (dentate gyrus) seem to undergo similar plasticity induced changes across age. Indeed this region is reportedly crucial for this task, and thus may require the same extent of plastic change for successive performance of the task regardless of age. Other regions showed age-dependent patterns in which the plasticity-induced changes observed by DTI lessened with age. This might indicate that these regions are already structurally developed and thus need no further changes. It is also possible, however, that it indicates an age-related weakening of the plasticity system in these regions.

### Technical Considerations

The current study relays on a statistical comparison between two or more groups. The comparison is done voxel-wise and therefore is susceptible to several pitfalls. One issue with such analysis refers to registration and normalization. This procedure may cause significant deformations of the original brain acquisition. As a consequence, the total signal of the brain may change [38] and influence the statistical analysis. However, in our study, such effects were not observed (data not shown).

Partial volume effect (PVE) can also contribute to biased statistics. This is especially true when acquiring non-cubic voxels (as in the current study). However, DTI necessitates high signal to noise ratio and therefore relatively large voxels should be used when the experiment is done *in-vivo*. To overcome the PVE, we choose to acquire non-cubic voxels in coronal orientation which have minimal PVE due to the geometry of the rat brain.

Additional issue is the multiple comparisons as the same statistical analysis is done on a large amount of voxels (in our case: 49,651 voxels). We have used the FDR method to correct for multiple comparisons as this method is conventionally used similar studies. However, one should consider, if the strength of the effect permits, to use a more strict corrections [39]. Nevertheless, the fact that whole brain analysis (with FDR correction) leads to changes in the hippocampus (the most reasonable region that should change following the behavioral procedure used in this work) and that these observations were replicated in an additional cohort (not shown), raise the validity of the observation beyond the multiple comparisons issue.

### Origins of the observed changes in diffusion MRI

In recent years, MRI studies of structural plasticity in the brain have shed some light on the process of neuroplasticity [20], [21], [22], [40]. Those studies indicated that prolonged training leads to a regional increase in cortical thickness and white matter organization. Although synaptogenesis is thought to be the main process accompanying plasticity in the adult brain, it seems reasonable to assume that a change in cortical thickness (as indicated by MRI) should be attributable to more than just an increase in the number of synapses. This is because the MRI signal is not highly sensitive to such fine changes. Diffusion MRI, like any MRI-related methodology, has the disadvantage of being nonspecific. In addition, each pixel incorporates the averaged water molecule signals from all tissue compartments, complicating making it difficult to correlate the changes observed on diffusion MRI with actual changes in microstructure. Further analysis and comparison with histology in the present study revealed a putative cellular basis for the observed DTI changes.

It is easier to interpret the changes observed in white matter than those seen in gray matter simply because there is better physical definition of FA in the white matter [31], [32]. Although it is tempting to assume that the axonal density is increased, there is not much evidence to support the generation of new axonal pathways in the mature brain, at least not within short time periods. However, changes in white matter structures, as measured by MRI, indicate that something occurs at the microstructural level when a new cognitive skill is learned [20], [21], [22]. Such structural changes might occur in existing axons, possibly because of changes in the myelination pattern in which myelin lamellas increase in number and become more tightly packed. Indeed, it was found that the radial diffusivity in these regions reduced (see supplementary material) which is known to be correlated with myelination status [41]. In addition, immunohistochemical analysis in the present study indeed revealed increased MBP staining, supporting the observed changes in diffusion MRI.

In gray matter regions, plasticity caused a decrease in both ADC and FA. The most significant finding that could account for these diffusion MRI observations was the astrocyte staining. Because MRI averages all cellular components, and because glial cells in general and astrocytes in particular represent the major cellular component in the brain, it is reasonable to assume that changes in diffusion MRI might be significantly influenced by glial morphology. Increased GFAP immunoreactivity was indeed observed in the dentate gyrus of the hippocampus in the learning group relative to the non-learning control group. An increase in GFAP staining indicates that the astrocytes are probably activated, with consequent changes in their shape, as well as an increase in the number of processes [8] and therefore an increase in their perimeter (see Fig. 3). Such changes can be expected to lead to an overall increase in tissue density and a concomitant reduction in ADC. Analysis of the principal diffusivities effect revealed that ADC reduction is mainly due to reduction in the radial diffusivity which is in agreement with the histological findings (increase in neural processes density) (see supplementary results). However, in the cingulate cortex, the FA decrease was a result of decrease in the axial diffusivity. Such observation could be correlated with staining to cellular markers that were not used in the present study (e.g. neurofilament stain).

### Future work and impact on plasticity studies

A process of neuroplasticity evidently accompanies the absorption of new information into the brain. Electrophysiological aspects of this process are well studied, and long-term potentiation and depression (LTP and LDP) are obviously the leading processes that contribute to plasticity. In recent years it was suggested that the observed electrophysiological changes are accompanied by activity-dependent remodeling of nerve tissue (neurons, their substructures, or their surrounding glial cells especially astrocytes). It is reasonable to postulate that these changes (electrophysiological and structural) are linked, and that the one type of change initiates and supports the other. In the present study we used MRI to investigate microstructural changes in the brain induced by plasticity, with the object of providing a means of neuroplasticity assessment to complement electophysiological measurements.

The ability to follow, *in vivo* and noninvasively, dynamic structural tissue changes induced by cognitive experience or training has far-reaching implications for neuroscience. As a complement to functional measurements, the use of diffusion imaging in general and DTI in particular to study structural plasticity can provide deeper insight into the affected regions, connectivity between regions, and timing of effects. Future studies should explore the time factor in these effects: how long do they last? What is the nature of their relationship to physiological measures such as LTP/LTD? What is the time course of regional plasticity changes? Do some regions undergo structural plasticity before others? These studies will help to unravel the mysteries of neuroplasticity, its time-dependent regional progression, and its relationship to behavioral variability among subjects. Finally, since MRI is a translational methodology, studies should be conducted in humans to find out whether the phenomena observed here in rats also occur in the human brain, and to define their regional patterns and time dependencies.

## Methods

### Ethics Statement

The study was approved by the Tel Aviv University Committee on Animal Care and Use and conducted according to the guidelines for research involving animals (permit number: L-08-032).

### Animals and experimental protocol

A total of **68** male Wistar rats, divided into three subgroups on the basis of age (26 aged 1 month, 22 aged 4 months, and 20 aged 12 months), comprised the experimental group (“learning group”, designated group L). All rats were maintained on a 12-h light/12-h dark cycle with access to food and water *ad libitum*. All underwent two MRI scans, 1 week apart, conducted in the light phase. Prior to scanning the rats were anesthetized with 1%–2% isoflurane in oxygen.

During the 5-day period between the two scans each rat in group L underwent daily training on a conventional behavioral learning and memory task in the Morris water maze [42], during which it learned the location of a hidden platform in a 120-cm-diameter pool. Four trials were conducted each day, with the rat placed in a different quadrant of the pool for each trial. The rat's main task was to memorize the location of the hidden platform based on spatial cues. An HVS video-tracking system (HVS Image Ltd., Hampton, UK) was used to record the time taken to reach the platform, as well as the time spent in each quadrant and the swimming speed.

Two control groups of 4-month-old rats underwent the same basic maintenance protocol as the rats in the experimental group, but received different treatment in the period between their two MRI scans. Rats in one control group (n = 10) were placed in the pool with a screen preventing them from perceiving spatial cues. Thus their task, though requiring comparable motor behavior to that in the experimental group, did not involve learning and memory. The rats in this “swimming-only” group (group S) were allowed to swim freely for a time period corresponding to the average time of the four daily trials performed by the rats in group L. This was done in order to equalize the swimming time of the two groups. The behavioral activity of the rats in group S was similarly recorded and the same analysis was applied to this group as to the experimental group.

Rats in the other control group, designated the “nonlearning group” (n = 14; group NL) did not undergo any behavioral manipulations but were simply kept in their cages between the two MRI scans, and were maintained there according to the same protocol as described for the other two groups.

After undergoing the second scan the rats were killed and their brains were removed and prepared for histology.

### Imaging

MRI was performed with a 7T MRI scanner (Bruker, Karlsruhe, Germany) with a 30-cm bore and a gradient strength of up to 400 mT/m. The MRI protocol included diffusion tensor imaging (DTI) acquisition with a diffusion-weighted (DW) spin-echo echo-planar-imaging (EPI) pulse sequence having the following parameters: TR/TE = 4000/25 ms, Δ/δ = 10/4.5 ms, four EPI segments, and 15 noncollinear gradient directions with a single b-value shell at 1000 s/mm^2^ and one image with a b-value of 0 s/mm^2^ (referred to as b0). Geometrical parameters were: 12 slices, each 1.2 mm thick (brain volume) and with in-plane resolution of 0.2×0.2 mm^2^ (matrix size 128×128; FOV 25.6 mm^2^). We have used an a-proportional voxel size on the anterior-posterior axis to increase signal-to-noise ratio. Obviously such voxel acquisition leads to partial volume effect (PVE) on that axis, however, in coronal rat brain acquisition, as used in the current study, PVE is relatively minor due to the morphology on the brain. The imaging protocol was repeated three times for signal averaging and to compensate for acquisition in which significant head motion was observed (see below). Each DTI acquisition took 4.5 min and the entire MRI protocol lasted about 20 min.

### MRI image analysis

Image analysis included DTI analysis of the DW-EPI images to produce the fractional anisotropy (FA), apparent diffusion coefficient (ADC), and radial and axial diffusivity indexed maps. The DTI analysis was implemented in Matlab (©Mathworks, USA) using in-house software. Because sporadic excessive breathing during DTI acquisition can lead to significant image motion artifacts that are apparent only in the slices sampled when the motion occurred, each image (for each slice and each gradient direction) was automatically screened, prior to DTI analysis, for motion artifacts. The signal profile along the phase-encoding direction was measured, and if the signal found outside the borders of the brain was significant the slice was omitted from the analysis. To compensate for this, DTI acquisition was repeated three times. Following the elimination of acquisition points with motion artifacts, the remaining acquisition points were corrected for linear (motion) and non-linear (eddy currents/susceptibility) artifacts using SPM2 (Wellcome Trust Centre for Neuroimaging, London, UK). It should noted, that the 4-shot EPI acquisition and the use of pre-defined EPI gradient pre-emphasis file resulted in negligible effect of eddy current and susceptibility which were only apparent at the lower edges of the brain (below the amygdala).

For statistical comparisons between rats, each rat brain volume was normalized with a template rat atlas allowing voxel-based statistics. All image transformations and statistical analyses described below were carried out using SPM2 (Wellcome Trust Centre for Neuroimaging, London, UK). The rat brain template was created from the data set of one representative rat that was registered with a digitized version of the Paxinos and Watson stereotactic atlas [43], and included a registered template b0 and FA images. Each rat data set was normalized to the template images. The normalization procedure included the following steps: (a) All b0 images initially underwent bias correction. This was done since data was acquired with quadrature surface coil designed for the rat head which caused slight intensity gradient (<15%) from the brain surface to the lower parts. (b) For each rat, the b0 image was co-registered with the b0 template (using a 6-parameter rigid-body transformation). The co-registration parameters were then applied on the different DTI indexed maps (FA, ADC, radial and axial diffusivities). Although, rigid body registration was applied on the raw-data, the b0 registration (affine) step was crucial for successful brain normalization (non-linear registration). (c) The registered FA maps were normalized to the FA template (having spatial resolution of 0.13×0.13×0.96 mm^3^) using a 12-parameter affine nonlinear transformation and 0.2 mm smoothing (which was used only for parameter estimation procedure and not smoothing of the output image). This was done to account for different brain shape and size between the animals. It should be noted that these differences are significantly smaller than those observed in the human brain but in order to remove this variability, normalization was necessary. Normalization was performed on the FA maps since they provide the most detailed visualization of brain structures and allow for more accurate normalization. The normalization parameters were then applied on all DTI indexed maps, including the FA, ADC, and radial and axial diffusivities. (d) The normalized indexed maps were smoothed with a 0.3-mm Gaussian kernel. To ensure that FA and ADC values were not affected significantly by the pre-processing steps, we have used the ‘nearest neighbour’ option following registration and normalization. In addition, the effects of normalization and registration on the standard deviations of the images are given in Figure S4. Following the abovementioned steps the standard deviation throughout the brain were similar, ensuring the validity of the statistical tests and verifying the comparison between regions.

### MRI statistical analysis

Following normalization, the following statistical tests were performed:

1. Mixed-design analysis of variance (ANOVA) for all 4-month-old rats in the three groups. Factorial ANOVA was performed with two factors: the three groups (L, S and NL) and the two scan times (with repeated measures on the second factor).
2. Mixed-design ANOVA for the experimental group in each of the three age subgroups (1, 4, and 12 months) Factorial ANOVA was performed with two factors: the three age subgroups and the two scan times (with repeated measures on the second factor).

For the two sets of comparisons, both main effects (scan time and group) and interaction (scan time vs. group) were obtained as statistical parametric maps. On all such maps, an effect was considered significant when the statistical threshold was *P*<0.05 (corrected for multiple comparisons using the false discovery rate (FDR) method). For purposes of illustration, however, we also report on regions that exceeded a lower threshold (see color maps in Figures 1, 4, 5 and S2, p<0.05, not corrected). The parametric maps are superimposed on an averaged FA map of all rats, and the atlas segmentation of brain regions (according to the atlas of Paxinos and Watson [43]) is outlined in blue. The observed changes were localized by inspection of the regions that exceeded the statistical threshold on the digitized atlas. Post-hoc analysis on the significant voxels was performed on clusters that exceeded the statistical threshold of *P*<0.05 (FDR corrected for multiple comparison).

### Immunohistochemistry

Five brains were selected for histological analysis from each of groups L and NL. Sections were stained with markers for the following cellular components: dendrites (stained with anti microtubule-associated protein 2, MAP2); synapses (with anti synaptophysin, SYN); myelin (with anti myelin basic protein, MBP); astrocytes (with anti glial fibrillary acidic protein, GFAP); and neurons (with anti neuronal nuclei, NeuN).

The perfused brains (4% paraformaldehyde) were cut by a sliding microtome (Leica SM 2000R, Wetzlar, Germany) into 30-µm coronal floating sections. Sections were washed twice with phosphate-buffered saline (PBS; 0.1 M, pH 7.4) and once with PBST (PBS containing 0.1% Triton X-100), and then blocked for 1 h in 20% normal donkey serum (Jackson ImmunoResearch Laboratories) in PBST. The sections were then transferred without rinsing into the primary antibody solution containing a rabbit polyclonal antibody directed against MAP2 (1∶1500; Chemicon International AB144), a mouse monoclonal antibody directed against SYN (1∶200; Sigma S5768), an anti GFAP antibody produced in rabbit (1∶100; Sigma G9269), or a mouse monoclonal antibody directed against MBP (1∶500; Covance SMI 99). Incubation for 48 h with the primary antibodies at 4°C was followed by three rinses with PBST. All sections were then incubated for 1 h with the secondary fluorescence antibody, donkey anti-rabbit or donkey anti-mouse (1∶1000; Alexa Flour, depending on the primary antibody), after which the sections were again rinsed three times in PBST, mounted on gelatin-coated slides, and coverslipped. Sections were inspected in a fluorescence microscope (Nikon Eclipse 80i DSFi1) and a confocal microscope (Zeiss LSM 510), and ROIs were photographed with a digital camera (Nikon Digital Sight) at different magnifications.

### Histological image analysis

Staining intensities were quantified by in-house software written in Matlab ©. Images (×10 magnification) were thresholded (same for all images) until background staining was minimized, and the intensity was then averaged over the image or ROI. Astrocyte shapes were analyzed geometrically with NIH-Image © on the ×20 magnification images. With this program, astrocytes were identified and numbered by the use of threshold and masking. The perimeter and area of each astrocyte were calculated. Student's *t*-test was used to compare group L with the two controls.

## Supporting Information

Figure S1 Performance in the Morris water maze. Normalized latency on each day of the water maze test, averaged over four trials per rat and for the entire group L, is shown for the different age subgroups. Latency was normalized to the average time for each age on the first day. As the test progressed the latency showed a clear decrease, which was much more pronounced for the younger age groups (1 and 4 months) than for the older one (12 months). Error bars represent the standard error for the entire group L. Compared to day 1, the improvement in latency was significant on all days and for all age subgroups (*P*<0.0001; Student's t-test).
(TIF)

Figure S2 Statistical parametric maps of the interaction between scan time and study group (groups L, S, and NL) for axial diffusivity (**a**) and radial diffusivity (**b**). The statistical maps (colored regions) are superimposed on an averaged FA map of all rats that were scanned, with the borders of the different anatomical regions outlined in blue (see Methods). Voxels that exceed a statistical threshold of *P*<0.05 are colored according to the threshold they exceeded (see color scale); those that did not exceed the threshold are not colored. Shown are only those regional clusters in which the most significant voxel exceeds a statistical threshold of *P*<0.005. These regions include the dentate gyrus (DG), entorhinal cortex (EC), piriform cortex (PC), amygdala (AMG), S1/S2 cortex (SC), corpus callosum (CC), visual cortex (VC) and entorhinal cingulum (Cg) in the axial diffusivity maps (**a**), and the VC, cingulate cortex (CG), dorsal hippocampal commissure (DHC), CC, EC, SC, AMG and PC in the radial diffusivity maps (**b**).
(TIF)

Figure S3 Histograms of astrocyte area analysis from GFAP staining in the hilus of the hippocampus. Note that after rats were trained in the water maze there was a decrease in the percentage of astrocytes whose area was smalland an increase in the percentage whose area was large.
(TIF)

Figure S4 Quality assessment of the registration and normalization procedures. Top row: mean FA of the entire group of rats for a representative slice (**a**), standard deviation map (**b**), and standard deviation divided by the mean (**c**) following registration only between the images. Bottom row: the same information but obtained after normalization: (**d**) mean FA, (**e**) standard deviation, (**f**) standard deviation divided by the mean. Note that registration was followed by significant misalignment between the images, resulting in high standard deviations for the border between white and gray matter and between gray matter and cerebrospinal fluid (**b, c**). After normalization these misalignments disappeared, as reflected by similarity of the standard deviations across the brain (**e, f**).
(TIF)

Text S1 (DOCX)
