# Supplementary figures and images for: Diffusion MRI of Structural Brain Plasticity Induced by a Learning and Memory Task
Source: PLoS One. 2011 Jun 20;6(6):e20678. doi: 10.1371/journal.pone.0020678 (PMC3119075; doi:10.1371/journal.pone.0020678)

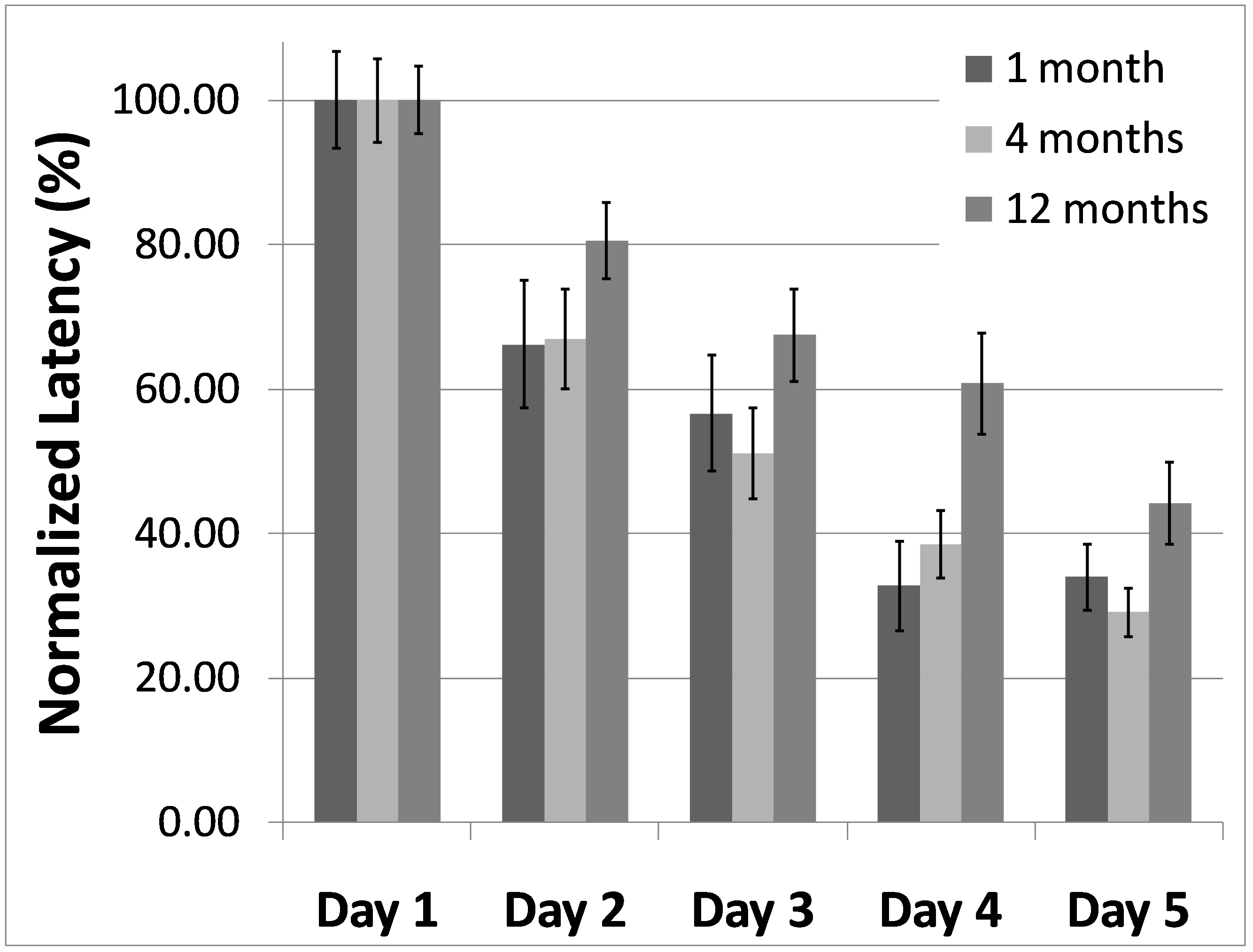

Supplement: Figure S1 — Performance in the Morris water maze. Normalized latency on each day of the water maze test, averaged over four trials per rat and for the entire group L, is shown for the different age subgroups. Latency was normalized to the average time for each age on the first day. As the test progressed the latency showed a clear decrease, which was much more pronounced for the younger age groups (1 and 4 months) than for the older one (12 months). Error bars represent the standard error for the entire group L. Compared to day 1, the improvement in latency was significant on all days and for all age subgroups (P<0.0001; Student's t-test). (TIF) [file pone.0020678.s001.tif]

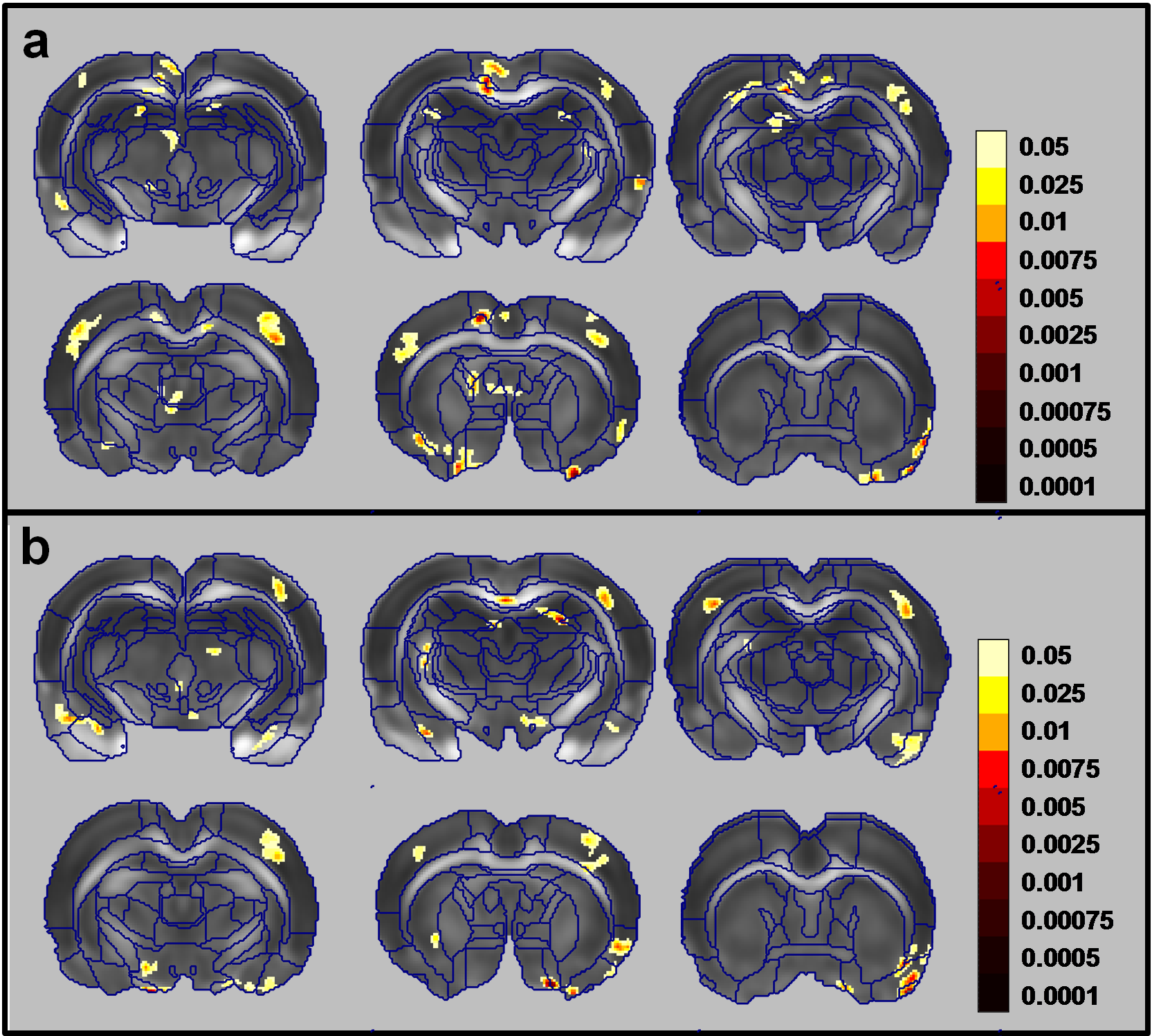

Supplement: Figure S2 — Statistical parametric maps of the interaction between scan time and study group (groups L, S, and NL) for axial diffusivity (a) and radial diffusivity (b). The statistical maps (colored regions) are superimposed on an averaged FA map of all rats that were scanned, with the borders of the different anatomical regions outlined in blue (see Methods). Voxels that exceed a statistical threshold of P<0.05 are colored according to the threshold they exceeded (see color scale); those that did not exceed the threshold are not colored. Shown are only those regional clusters in which the most significant voxel exceeds a statistical threshold of P<0.005. These regions include the dentate gyrus (DG), entorhinal cortex (EC), piriform cortex (PC), amygdala (AMG), S1/S2 cortex (SC), corpus callosum (CC), visual cortex (VC) and entorhinal cingulum (Cg) in the axial diffusivity maps (a), and the VC, cingulate cortex (CG), dorsal hippocampal commissure (DHC), CC, EC, SC, AMG and PC in the radial diffusivity maps (b). (TIF) [file pone.0020678.s002.tif]

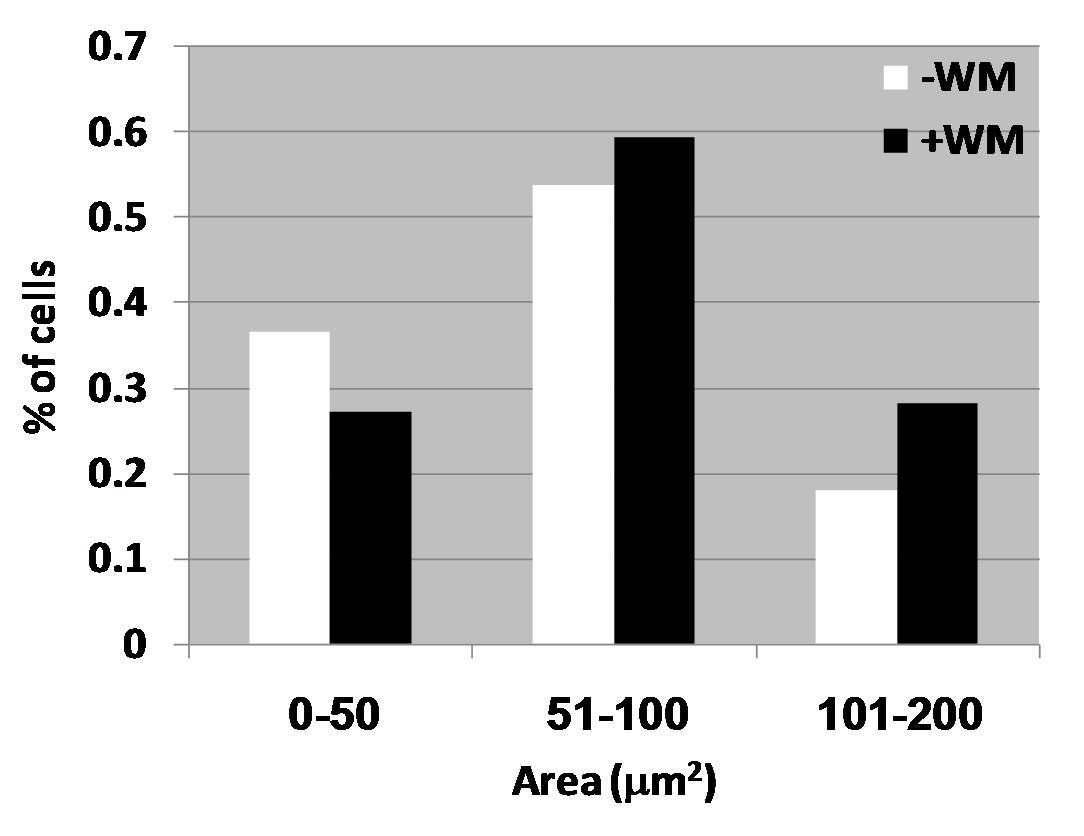

Supplement: Figure S3 — Histograms of astrocyte area analysis from GFAP staining in the hilus of the hippocampus. Note that after rats were trained in the water maze there was a decrease in the percentage of astrocytes whose area was smalland an increase in the percentage whose area was large. (TIF) [file pone.0020678.s003.tif]

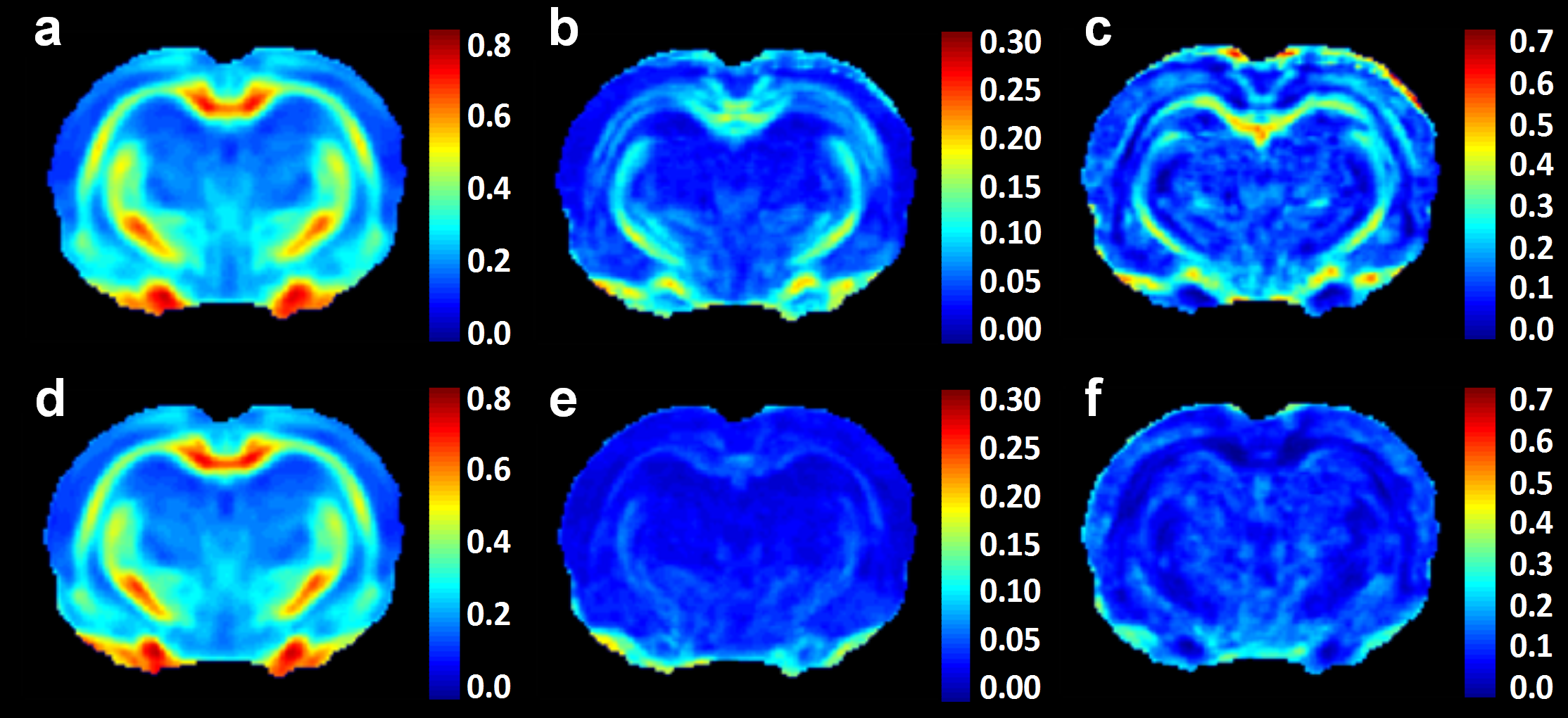

Supplement: Figure S4 — Quality assessment of the registration and normalization procedures. Top row: mean FA of the entire group of rats for a representative slice (a), standard deviation map (b), and standard deviation divided by the mean (c) following registration only between the images. Bottom row: the same information but obtained after normalization: (d) mean FA, (e) standard deviation, (f) standard deviation divided by the mean. Note that registration was followed by significant misalignment between the images, resulting in high standard deviations for the border between white and gray matter and between gray matter and cerebrospinal fluid (b, c). After normalization these misalignments disappeared, as reflected by similarity of the standard deviations across the brain (e, f). (TIF) [file pone.0020678.s004.tif]
