# Supplementary material for: Diffusion MRI of Structural Brain Plasticity Induced by a Learning and Memory Task
Source: PLoS One. 2011 Jun 20;6(6):e20678. doi: 10.1371/journal.pone.0020678 (PMC3119075; doi:10.1371/journal.pone.0020678)
Supplement: Text S1 — (DOCX) [file pone.0020678.s005.docx]

**Plasticity effect on the principal diffusivities.** Effects on ADC and FA are a consequence of changes in axial and radial diffusivities. Parametric interaction maps of the axial and the radial diffusivities are summarized in Figure S2. The regional localizations of significant interactions in axial and in radial diffusivities are the same as those obtained in the ADC and FA maps. In general, an increase in FA was a result of decrease in the radial diffusivity, an increase in ADC was a result of increase in radial diffusivity, and a decrease in ADC was a result of decrease in both axial and radial diffusivities.
